# Impact of PET acquisition durations on image quality and lesion detectability in whole-body ^68^Ga-PSMA PET-MRI

**DOI:** 10.1186/s13550-017-0261-8

**Published:** 2017-02-06

**Authors:** Benjamin Noto, Florian Büther, Katharina Auf der Springe, Nemanja Avramovic, Walter Heindel, Michael Schäfers, Thomas Allkemper, Lars Stegger

**Affiliations:** 1 Department of Nuclear Medicine, University Hospital Münster, Albert-Schweitzer-Campus 1, 48149 Münster, Germany; 2 European Institute for Molecular Imaging, University of Münster, Münster, Germany; 3 Institute for Clinical Radiology, University Hospital Münster, Münster, Germany

**Keywords:** PET-MRI, PSMA, Prostate cancer, Halo artefacts, Attenuation correction, Scatter correction

## Abstract

**Background:**

While ^68^Ga-PSMA PET-MRI might be superior to PET-CT with regard to soft tissue assessment in prostate cancer evaluation, it is also known to potentially introduce additional PET image artefacts. Therefore, the impact of PET acquisition duration and attenuation data on artefact occurrence, lesion detectability, and quantification was investigated.

To this end, whole-body PET list mode data from 12 patients with prostate cancer were acquired 1 h after injection of 2 MBq/kg [^68^Ga]HBED-CC-PSMA on a hybrid PET-MRI system. List mode data were further transformed into data sets representing 300, 180, 90, and 30 s acquisition duration per bed position. Standard attenuation and scatter corrections were performed based on MRI-derived attenuation maps, complemented by emission-based attenuation data in areas not covered by MRI. A total of 288 image data sets were reconstructed with varying acquisition durations for emission and attenuation data with and without scatter and prompt gamma correction, and further analysed regarding image quality and diagnostic performance.

**Results:**

Decreased PET acquisition durations resulted in a significantly increased incidence of halo artefacts around kidneys and bladder, decreased lesion detectability and lower SUV as well as markedly lower arm attenuation values: Halo artefacts were present in 5 out of 12 cases at 300-s duration, in 6 at 180 s, in 10 at 90 s, and in 11 cases at 30 s. Using attenuation data of the 300 s scans restored artefact occurrence to the original 300-s level. Prompt gamma correction only led to small improvements in terms of artefact occurrence and size. Of the 141 detected lesions in the 300-s images one lesion was not detected at 180 s, 28 at 90 s, and 64 at 30 s. Using the 300-s attenuation map decreased non-detectability of lesions to zero at 180 s, 9 at 90 s, and 52 at 30 s. Attenuation maps at 90 and 30 s demonstrated markedly lower mean arm attenuation values (0.002 cm^-1^) than those at 300 s (0.084 cm^-1^), and 180 s (0.062 cm^-1^).

**Conclusions:**

Short acquisition durations of less than 3 minutes per bed position result in unacceptable image artefacts and decreased diagnostic performance in current whole-body ^68^Ga-PSMA PET-MRI and should be avoided. Increased image noise and imperfections in generated attenuation maps were identified as a paramount cause for image degradation.

**Electronic supplementary material:**

The online version of this article (doi:10.1186/s13550-017-0261-8) contains supplementary material, which is available to authorized users.

## Background

Cells of prostate cancer, the most frequent cancer in men worldwide [1], usually show an overexpression of the membrane-bound metallopeptidase prostate specific membrane antigen (PSMA) [2]. Several radioactively-labelled PSMA ligands have been developed for prostate cancer imaging with positron emission tomography (PET) and single photon emission computed tomography (SPECT) [3–5]. Of these, [^68^Ga]-PSMA-HBED-CC (^68^Ga-PSMA) has found the most widespread application for use with PET-CT (PET combined with computer tomography). There is mounting evidence that ^68^Ga-PSMA PET-CT could become a new standard for imaging of prostate cancer and its metastases [6–8].

The recently introduced integrated PET-MRI systems promise further advances in multimodal imaging of prostate cancer by providing better soft tissue contrast, additional parameters such as diffusion and perfusion, and a reduced radiation exposure compared to PET-CT. Consequently, a current study suggests a superiority of ^68^Ga-PSMA PET-MRI for the detection of prostate cancer lesions compared to PET-CT [9].

It has to be noted, however, that ^68^Ga-PSMA PET-MRI images can exhibit areas of artificially reduced apparent tracer uptake (also known as “halo artefacts”) in the vicinity of “hot” structures with high tracer uptake (especially kidneys and bladder) in scatter-corrected images [10–12]. Critically, tumour manifestations within these areas might be non-detectable. Additional images, reconstructed without scatter correction, may alleviate this problem to a certain degree [12].

Further insight into the cause of these artefacts and subsequent optimization of imaging protocols are warranted as a consequence in order to minimise the adverse effect on diagnostic accuracy. A recent study by Lütje et al. investigated the influence of acquisition time per bed position on the degree of halo artefacts for a single-bed position (pelvis) and at delayed imaging time points (3 h after injection) [13]. The results of this study show a degradation of image quality with decreasing PET acquisition duration and support an optimal scan duration of 4 min per bed position.

Scatter correction algorithms rely on an accurate attenuation map. Investigating the influence of the implementation of attenuation correction on the aforementioned halo artefacts should help to provide valuable insights into this problem. MR-based attenuation correction is not straightforward, since the image information provided by MRI is not directly related to tissue density with respect to ionizing radiation as in PET or CT. The standard procedure implemented for the Siemens Biograph mMR scanner (Siemens Healthineers, Erlangen, Germany) is based on MR-based segmentation of different tissue classes (air, lung, fat, water; ±bone for brain imaging) and voxel-wise application of typical attenuation values. Besides known issues in bone attenuation correction [14], attenuation maps of the arms of patients represent a major challenge. Due to limited space in the scanner bore and prolonged scanning durations, patients are usually positioned with their arms beside their bodies, while the standard procedure for PET-CT is scanning with arms elevated over the head. The transverse MRI field of view in the current implementation of the Siemens mMR sequence for attenuation correction is too small to completely include the arms in most cases resulting in truncated attenuation maps [15]. The missing portion of the attenuation map is derived from the PET emission data [16]; therefore, PET scan duration could indirectly influence the quality of the attenuation map and finally the quality of the reconstructed images.

Furthermore, it has been suggested that scattered prompt gamma photons of 1077 keV emitted during a fraction of decays of ^68^Ga may contribute to the occurrence of halo artefacts in ^68^Ga-PSMA PET-CT [17]; however, systematic investigations of this issue in PET-MRI have not yet been conducted.

In this study, we therefore investigated the influence of PET acquisition duration on the quality of both emission as well as attenuation data in whole-body ^68^Ga-PSMA PET-MRI obtained 1 h after tracer injection and how this affects image artefacts, lesion detectability, and quantification.

## Methods

### Patient data and preparation

All patients included into this study underwent PET-MRI imaging with a clinical indication, from June to September 2016. The clinically acquired PET and MRI data were retrospectively analysed in accordance to local institutional and ethical guidelines.

Data from twelve male patients (age: 65.3 ± 9.3 years, body mass: 86.8 ± 9.1 kg) who were referred for ^68^Ga-PSMA PET-MRI, either for initial staging of prostate cancer (*n* = 3) or for restaging due to biochemical recurrence (*n* = 9), were included in this study. Approximately 2 MBq [^68^Ga]-PSMA-HBED-CC per kg body weight (range: 144–198 MBq) were injected intravenously, followed by a 20 ml saline flush, 65 ± 9 min (range: 54–82 min) before the start of the PET scan. Patients were told to void their bladder before the scan.

### Data acquisition

All data were acquired on a combined PET-MRI system capable of simultaneous PET and MRI acquisition (Biograph mMR, Siemens Healthineers, Erlangen, Germany; NUMARIS/4 software version B20P). Patients were positioned supine with arms lying next to the body. Head/neck and body surface coils were used for MR imaging. Imaging started with a standard two-point Dixon sequence for MR-based attenuation correction. All voxels in the field of view were classified into one of the four tissue compartments air, lung, fat, and water in order to provide an attenuation map of the volume covered by MRI. Subsequent PET imaging covered the whole-body from the head to the knee level, resulting in five to seven bed positions. For all bed positions PET data were collected for 5 min in “list mode” acquisition mode, in which individual counts are saved for later sorting and image reconstruction. During and after PET acquisition diagnostic, MR imaging was performed; MRI data were only used in this study to distinguish tumour lesions from physiological activity, e.g., in the urogenital tract, by consensus reading of two nuclear medicine and two diagnostic radiology experts (one in each group board certified with >15 years of clinical experience).

### PET image reconstruction

All relevant acquisition data (PET list mode data, MR-derived attenuation map) were transferred to a separate workstation for offline data processing. Whole-body list mode PET data were reconstructed using the offline sorting and reconstruction programs included in the e7 toolbox (Siemens Healthineers, Erlangen, Germany; version VA20). To this end, every list mode file was first binned into sinograms. In order to investigate different acquisition durations, several sinogram sets were constructed by first using all data from the 300-s scan, then with decreasing statistics by only taking into account the first 180, 90, and 30 s of list mode data, respectively, as described previously by Armstrong et al. [18]. Attenuation maps (AM) were generated from MR data (standard Dixon sequence-based segmentation of air, lungs, fat-, and water-like tissue). Between shoulder and pelvis, where MRI coverage of the arms is incomplete, maps were automatically complemented by attenuation data derived from the PET emission data using the maximum likelihood reconstruction of activity and attenuation (MLAA) algorithm [16]. Since the sinogram data are used for both the emission data and for MLAA-based attenuation correction (AC), and hence also for scatter correction (SC), several different combinations of emission/AM timings are possible.

**Fig. 1:**
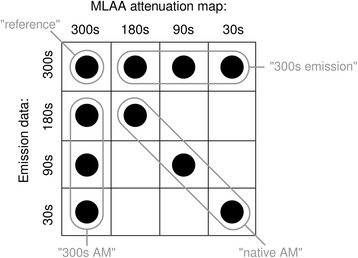
PET reconstructions performed in this study, combining different emission, and attenuation data sets

For each patient the following ten image data sets were reconstructed from these sinogram data (Fig. 1):

1. Reference: sinograms obtained from the full 300 s were used for both emission data and MLAA-based attenuation and scatter correction (one data set);
2. Emission and AM reduced (“native AM”): sinogram data with reduced acquisition duration were used in parallel for the emission data and for MLAA-based attenuation and scatter correction (three data sets for 180, 90, 30 s);
3. Emission-only reduced (“300-s AM”): sinograms with reduced time duration were only used for the emission data while the complete 300-s sinogram data (as representing arguably the best estimate of arm attenuation) were used for MLAA-based attenuation and scatter correction (three data sets);
4. AM-only reduced (“300-s emission”): sinograms with reduced time duration were only used for MLAA-based attenuation and scatter correction while the complete 300-s sinogram data were used for the emission data (three data sets).

The image data under (2) reflect the clinical situation with reduced acquisition times, while the image data under (3) and (4) provide insight into the relative contribution of the emission data and the attenuation maps. All PET data were calibrated, normalised, and corrected for attenuation, random and scattered coincidences. Distributions of scattered events for scatter correction were calculated using the standard e7 scatter correction algorithm based on the respective emission data and the determined MLAA-based attenuation maps. Scaling of these distributions was performed by tail-fitting to the measured emission data outside the apparent body of the patient as determined from the attenuation map (“relative scaling”).

In order to determine the influence of prompt gamma photons on halo artefact occurrence, reference and native AM data were additionally reconstructed with the prompt gamma correction (PGC) algorithm for ^68^Ga implemented in the e7 tools which is currently not available on the clinical PET-MRI system.

Whole-body image reconstructions were performed using a standard ordinary Poisson ordered subsets expectation maximization algorithm (OP-OSEM) with 3 iterations and 21 subsets. A Gaussian filter with 5 mm full-width-half-maximum was applied after reconstruction. Reconstructed PET images consisted of 344 × 344 voxels in the axial slices, each with dimensions of 2 × 2 × 2 mm^3^.

For further testing of the assumption that additional images reconstructed without scatter correction can alleviate some of the problems caused by the halo artefacts, additional images with attenuation correction but without scatter and prompt gamma correction were reconstructed for all of the above scenarios. In total, 24 image data sets per patient (288 altogether) were reconstructed for subsequent analysis.

### Image analysis

All PET images were analysed visually by two experienced nuclear medicine professionals (physician and physicist) with respect to the presence and location of “halo artefacts” and with respect to the detectability of tumour lesions. Additionally, tracer uptake in the scatter-corrected images was quantified as maximum standardized uptake values (SUV_max_). Finally, the volume of the arms added to the MRI-derived attenuation map by the emission-based MLAA algorithm was determined for every image data set in the pelvic bed position, together with a typical attenuation value found in a portion of the forearm added to the MRI-based attenuation map by the MLAA method. All analyses were undertaken by using the AMIDE image analysis software (Version 1.0.1, amide.sourceforge.net).

#### Halo artefacts

Halo artefacts, presenting in the scatter-corrected images as apparently hypointense regions around the kidneys and the bladder (i.e., the organs with a very high tracer accumulation), were classified with respect to location and quantity: 0: no visible artefact; 1: halo around either kidneys or bladder; 2: halos around kidneys and bladder.

#### Tumour detectability

The detectability of PSMA-positive tumour lesions that were visible in the reference images reconstructed using 300-s acquisition data were classified for all image data sets with reduced count statistics (reduced for emission data, attenuation correction or both, with and without scatter correction) according to the following scheme: 0: not detectable; 1: equivocal; 2: discernible; 3: clearly visible. Since both image data sets, with and without scatter correction (+SC and -SC), are available for image interpretation in clinical routine, a composite score was calculated as the maximum score from both image data sets.

#### Standardized uptake values (SUV)

Maximum standardized uptake values (SUV_max_) were calculated for every tumour lesion with a detectability score >0 in the scatter-corrected images at all scan durations as follows:

$$ S U{V}_{max}=\frac{c_{max}\cdot dcf\cdot m}{A_0}, $$

with *c*_*max*_ denoting the maximum ^68^Ga-PSMA radioactivity concentration in the respective lesion, *m* the body mass of the patient, *A*_0_ the injected amount of radioactivity at time of injection, and *dcf* the decay correction factor between the time of tracer injection and the start time of the PET scan.

As prompt gamma correction is currently not available for clinical reconstructions, tumour detectability and SUV determination was not performed on the images reconstructed using the prompt gamma correction algorithm of the e7 tools.

In order to assess possible relations between tracer uptake and halo occurrence, SUV_mean_ of the brightest kidney and of the bladder as defined by a volume of interest based on 50% of SUV_max_ were determined and correlated to halo occurrence around the respective organ. This analysis was performed on the original PET data set comprising 300-s acquisition duration per bed position.

#### Attenuation map alterations caused by MLAA

The pelvic bed positions were analysed to assess the influence of decreasing emission data statistics on MLAA-based arm completion in the computed attenuation maps. This was done by determining the total volume of arm voxels added to the Dixon sequence-derived attenuation map by MLAA with attenuation values higher than 0.005 cm^-1^. Additionally, the mean attenuation value of a representative spherical volume-of-interest (VOI) in the right forearm (diameter: 20 mm) was determined.

### Statistics

Data are presented as mean ± standard deviation. Significant differences were tested by paired two-tailed Wilcoxon signed rank tests (for detectability scores and SUV) using the statistical toolbox in MATLAB (version 2013b; MathWorks, Natick, USA). A Holm-Bonferroni correction for pairwise comparisons was applied to compensate for the growth of the family-wise error rate when performing multiple tests. The family-wise error rate was set to 0.05.

### Case presentation

Finally, we present a typical case to highlight the clinical impact of different acquisition durations on ^68^Ga-PSMA PET image quality in PET-MRI.

## Results

### Halo artefacts

**Fig. 2:**
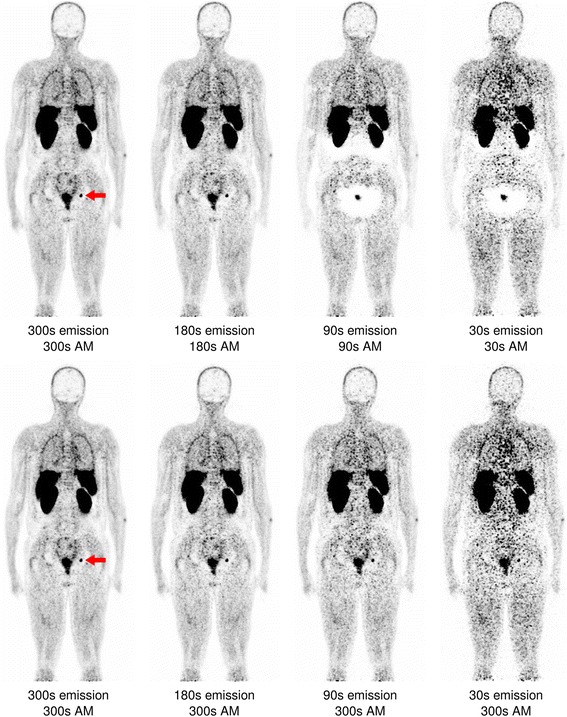
Coronal slice of a whole-body PSMA-PET reconstructed with varying acquisition durations for both emission data and attenuation map creation (“native AM”, *upper row*), and varying acquisition durations only for the emission data while the attenuation map was derived from the full 300-s data set (“300-s AM”, *lower row*). The red arrow marks a lymph node metastasis, clearly visible in the high-statistics 300- and 180-s images but undetectable in the 90 and 30-s scans using native AM

**Fig. 3:**
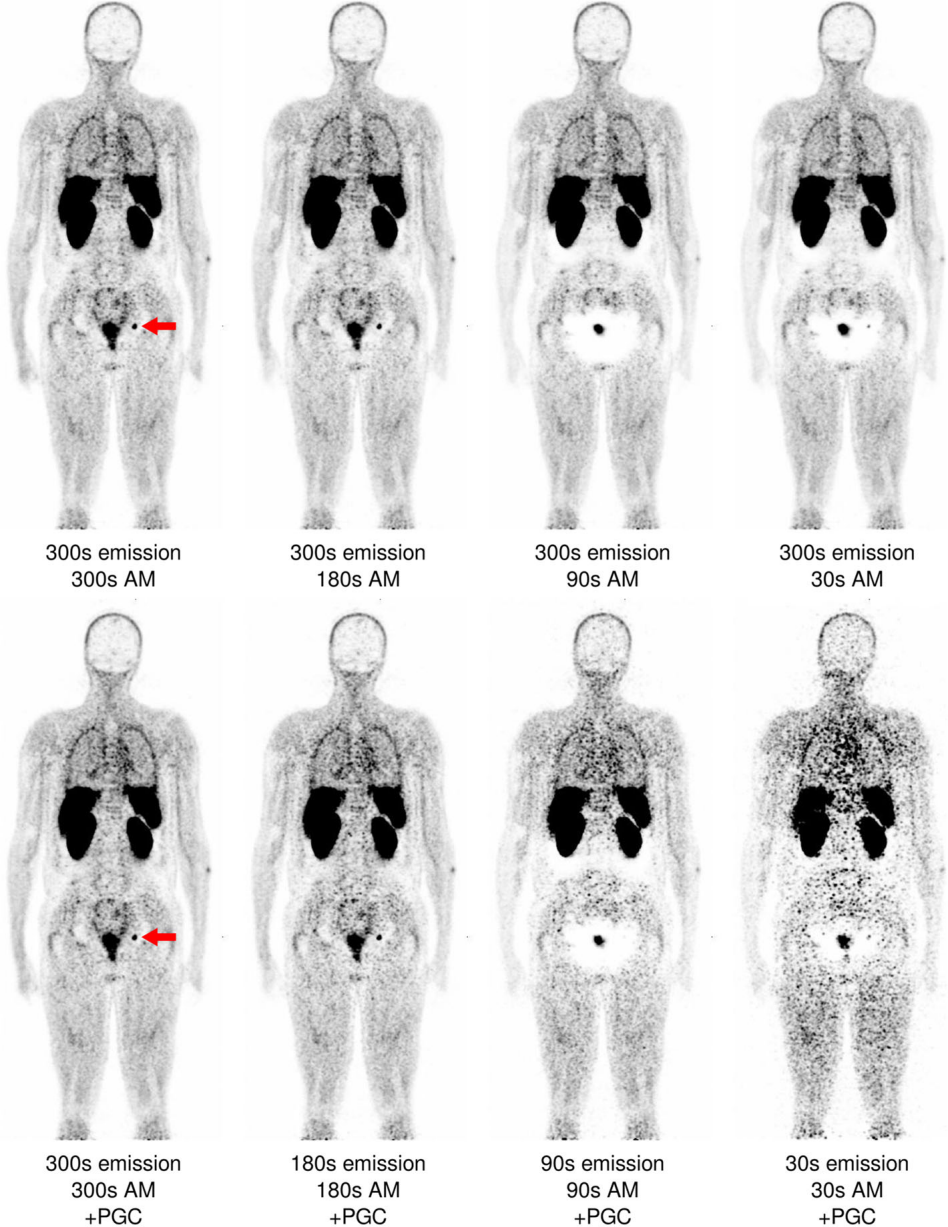
Coronal slice of a whole-body PSMA-PET (identical to Fig. 2) reconstructed with the full 300-s emission data but with a decreasing count statistics for attenuation map generation (“300-s emission”, *upper row*), and reconstructed with varying acquisition durations for both emission data and attenuation map creation as well as prompt gamma correction (“native AM + PGC”, *lower row*). The lymph node again is not visible at 180 s but weakly reappears in the 30-s data sets

In native scatter- and attenuation-corrected images, distinct halo artefacts around the kidneys and/or bladder were seen in five out of 12 cases (41.7%) for 300-s images, in 6 for 180 s (50.0%), in 10 for 90 s (83.3%), and in 11 cases for 30-s images (91.7%; Table 1, Fig. 2, Additional file 1), indicating a marked increase in the number of halo artefacts with decreasing count statistics. Mean halo scores (± standard deviation) amounted to 0.50 ± 0.67 for 300 s, 0.67 ± 0.78 for 180 s, 1.67 ± 0.78 for 90 s, and 1.67 ± 0.65 for 30 s. Differences between 300 s and 90 s/30 s as well as differences between 180 s and 90 s/30 s were significant. Interestingly, when using the attenuation map derived from 300-s emission data for AC and SC, no such increase in halo artefacts with reduction of acquisition duration was observed, and halo scores remained on the level of 300-s emission data (Fig. 2). In contrast, emission data obtained from the full 300-s data set reconstructed with attenuation maps of decreased scanning durations again resulted in increasing artefact numbers (Fig. 3), demonstrating the profound effect of MLAA-based arm completion on halo artefact appearance. Native images reconstructed using prompt gamma correction had mean halo scores of 0.50 ± 0.67 for 300 s, 0.67 ± 0.78 for 180 s, 1.67 ± 0.78 for 90 s (identical to the scores of the images reconstructed without PGC), and 1.25 ± 0.75 for 30 s, demonstrating a limited effect of this correction on halo occurrence, although artefact sizes seemed to be slightly smaller in the images corrected for prompt gammas (Fig. 3).

**Table 1:** Number and locations of halo artefacts in a total of 12 whole-body ^68^Ga-PSMA PET-MRI scans

	Native AM		300-s AM		300-s emission		Native AM, +PGC	
Acquisition duration (s)	Kidney halo	Bladder halo	Kidney halo	Bladder halo	Kidney halo	Bladder halo	Kidney halo	Bladder halo
300	4	2	4	2	4	2	4	2
180	6	2	4	2	6	2	6	2
90	10	10	4	2	10	10	10	10
30	11	9	3	2	11	9	10	5

### Lesion detectability

Altogether, 141 lesions were identified in 12 patients (range: 0–35 lesions per patient): 98 bone metastases, 33 lymph node metastases, 8 lesions of the prostate gland or seminal vesicle and 2 liver metastases. All lesions were located in bed positions where MLAA-based completion of the attenuation maps were performed (i.e., from shoulder to pelvic regions).

**Fig. 4:**
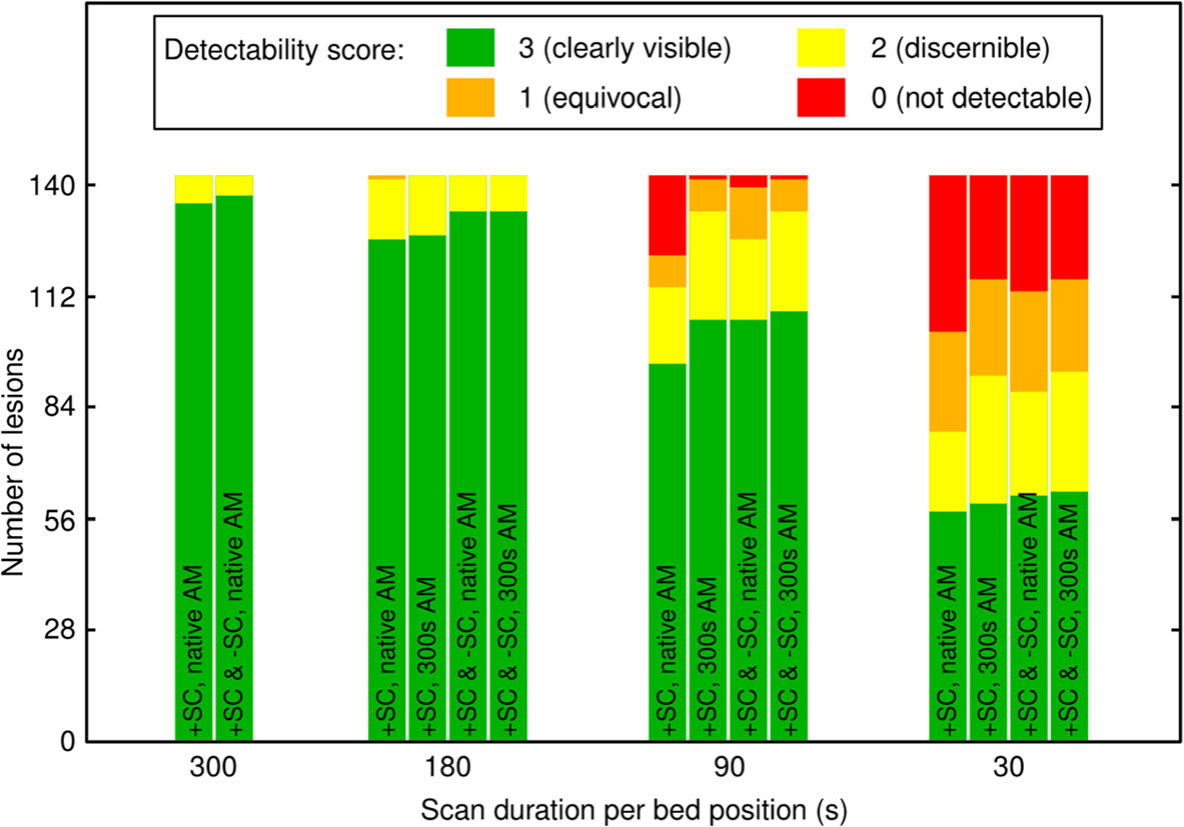
Number of lesions and detectability scores of 141 PSMA-PET-positive lesions for different reconstruction types and acquisition durations

In the scatter-corrected images all except one of the unequivocally detectable lesions (score value ≥2) in the reference 300-s images were also detected in the native 180-s images (0.7%); however, 28 lesions were undetectable in 90-s images (19.9%), and 64 in 30-s images (45.4%; Fig. 4, Additional file 2). When using the 300-s attenuation map for AC and SC, only 9 lesions were missed in the 90-s images (6.4%), and 52 in the 30-s images (36.9%).

In the images reconstructed without scatter correction, all lesions were detectable in the 300-s data set. However, three lesions were undetectable in the 180-s images (2.1%), 22 in 90-s (15.6%) and 66 in 30-s images (46.8%). Both data sets, with and without scatter correction (+SC and -SC) taken together, yielded zero undetectable lesions when going from 300-s to 180-s but 16 undetectable lesions in the 90-s (11.3%) and 54 in the 30-s images (38.3%) in the case of native attenuation maps, and 9 undetectable lesions in the 90-s (6.4%) and 49 in the 30-s images (34.8%) in the case of using the 300-s attenuation map (Fig. 4).

The mean detectability scores in the scatter-corrected images amounted to 2.95 ± 0.22 for 300-s images, 2.88 ± 0.35 for 180 s, 2.33 ± 1.09 for 90 s, and 1.67 ± 1.26 for 30-s images. Every difference was found to be significant. Using the 300-s attenuation map for AC and SC, detectability scores improved to 2.89 ± 0.31 for 180 s, 2.67 ± 0.62 for 90 s and 1.88 ± 1.15 for 30-s images, respectively. Only the latter two values were found to be significantly different from the original value. This improvement was caused by halo artefact suppression, thus lesions previously masked by halos became detectable. The residual loss in detectability was most likely caused by increased image noise.

Mean detectability scores in the non-scatter-corrected images amounted to 2.91 ± 0.29 for 300-s images, 2.85 ± 0.41 for 180 s, 2.47 ± 0.82 for 90 s and 1.60 ± 1.20 for 30-s images; each score value was found to be significantly different from the others. Both data sets with and without scatter correction (+SC and -SC) taken together yielded 2.96 ± 0.19 for 300-s images, 2.94 ± 0.25 for 180 s, 2.61 ± 0.74 for 90 s, and 1.84 ± 1.19 for 30-s images in the case of native attenuation maps, and 2.94 ± 0.25 for 180 s, 2.69 ± 0.61 for 90 s, and 1.91 ± 1.16 for 30-s images in the case of using the 300-s attenuation map. All differences in detectability scores were found to be significant in both cases, except the differences between 300 and 180 s.

### Standardized uptake values (SUV)

**Fig. 5:**
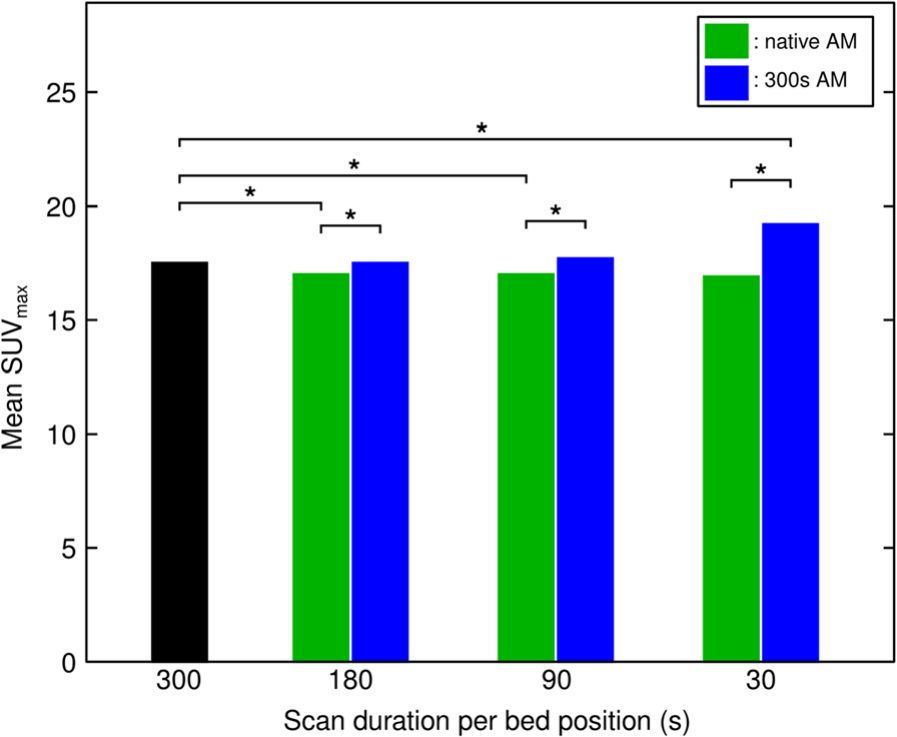
Mean SUV_max_ for different PET acquisition durations and AM settings. An *asterisk* denotes significant differences

The number of lesions with a detectability score >0 (at least equivocally detectable) at all scan durations amounted to 102. Among these, reduction of acquisition duration from 300 to 180 or 90 s (for both emission data and attenuation/scatter correction likewise) led to a significant drop in SUV_max_ (17.6 ± 17.4 for 300s, 17.1 ± 17.4 for 180s, 15.9 ± 16.8 for 90-s images; Fig. 5, Additional file 3). For 30-s images mean SUV_max_ was 17.0 ± 17.8, a slight increase from the 90-s images. When using the 300-s attenuation map for AC and SC, the opposite effect was observed: Reduction in acquisition duration led to an increase in mean SUV_max_ (17.6 ± 17.8 for 180 s, 17.8 ± 17.9 for 90 s, and 19.3 ± 19.3 for 30-s images).

**Fig. 6:**
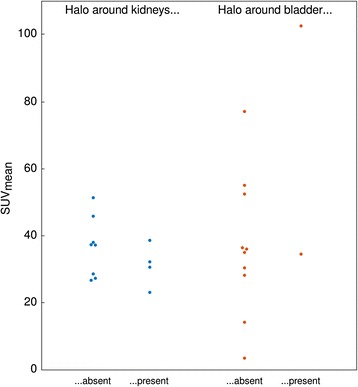
Halo artefact occurrence and SUV_mean_ of high-contrast organs in the reference 300-s images

No apparent relation was found between SUV_mean_ of either kidneys or bladder and halo artefact occurrence (Fig. 6 and Additional file 4).

### Attenuation maps

**Fig. 7:**
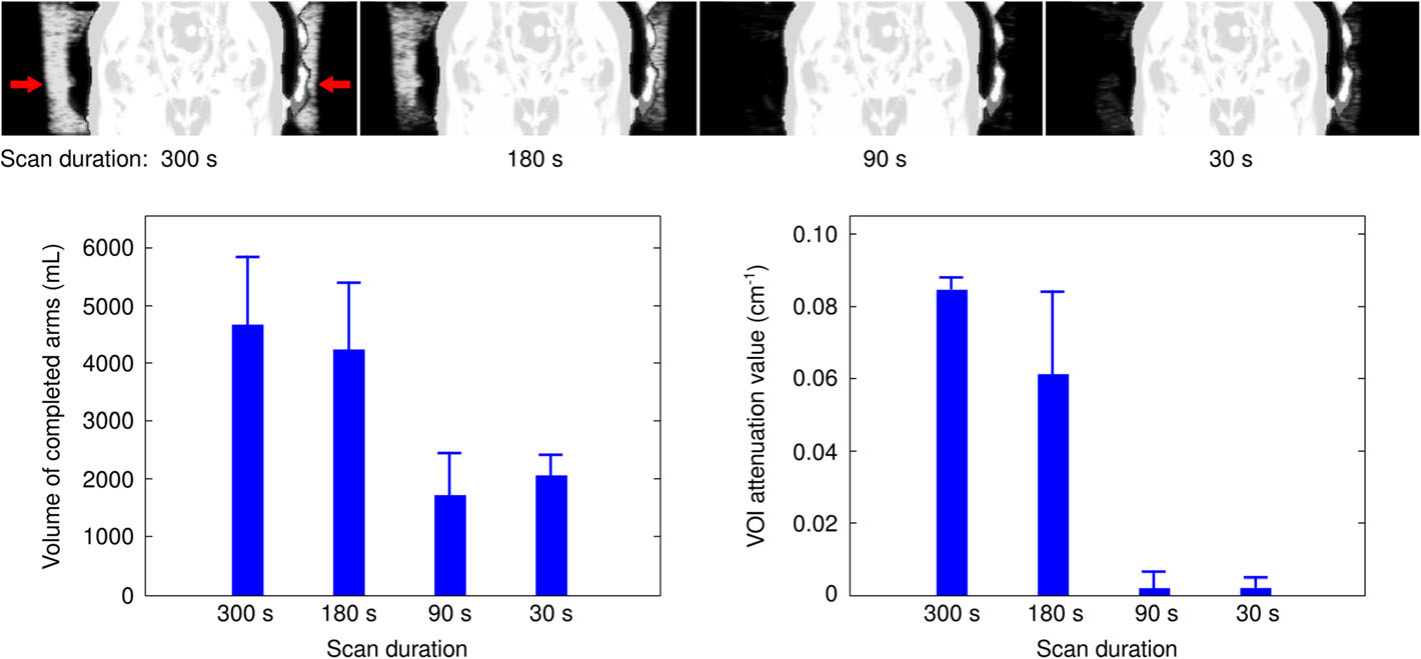
Example of MLAA-derived arm attenuation completion at different acquisition durations (coronal slices, *top row*). The volume of completed arms generally decreases with decreasing durations (*bottom left*). A similar trend is seen in the attenuation map values in a VOI inside the right arm (20 mm diameter, *bottom right*)

Reduction of acquisition time showed a significant impact on arm completion in the MLAA-based attenuation map (Fig. 7 and Additional file 5). The volume of completed arms in the pelvic bed position decreased from 4648 ± 1180 mL for 300-s images to 4109 ± 1314 mL for 180 s and 1745 ± 688 mL for 90 s images. For the 30-s images, the additional arm volume increased again to 2055 ± 375 mL. The mean VOI value of the arm attenuation map dropped from 0.084 ± 0.004 cm^-1^ for 300-s images to 0.062 ± 0.023 cm^-1^ for 180 s and 0.002 ± 0.004 cm^-1^ for 90-s images (water attenuation value for comparison: 0.096 cm^-1^). Further reduction to 30-s acquisition time resulted in a mean value of 0.002 ± 0.003 cm^-1^.

### Case presentation

**Fig. 8:**
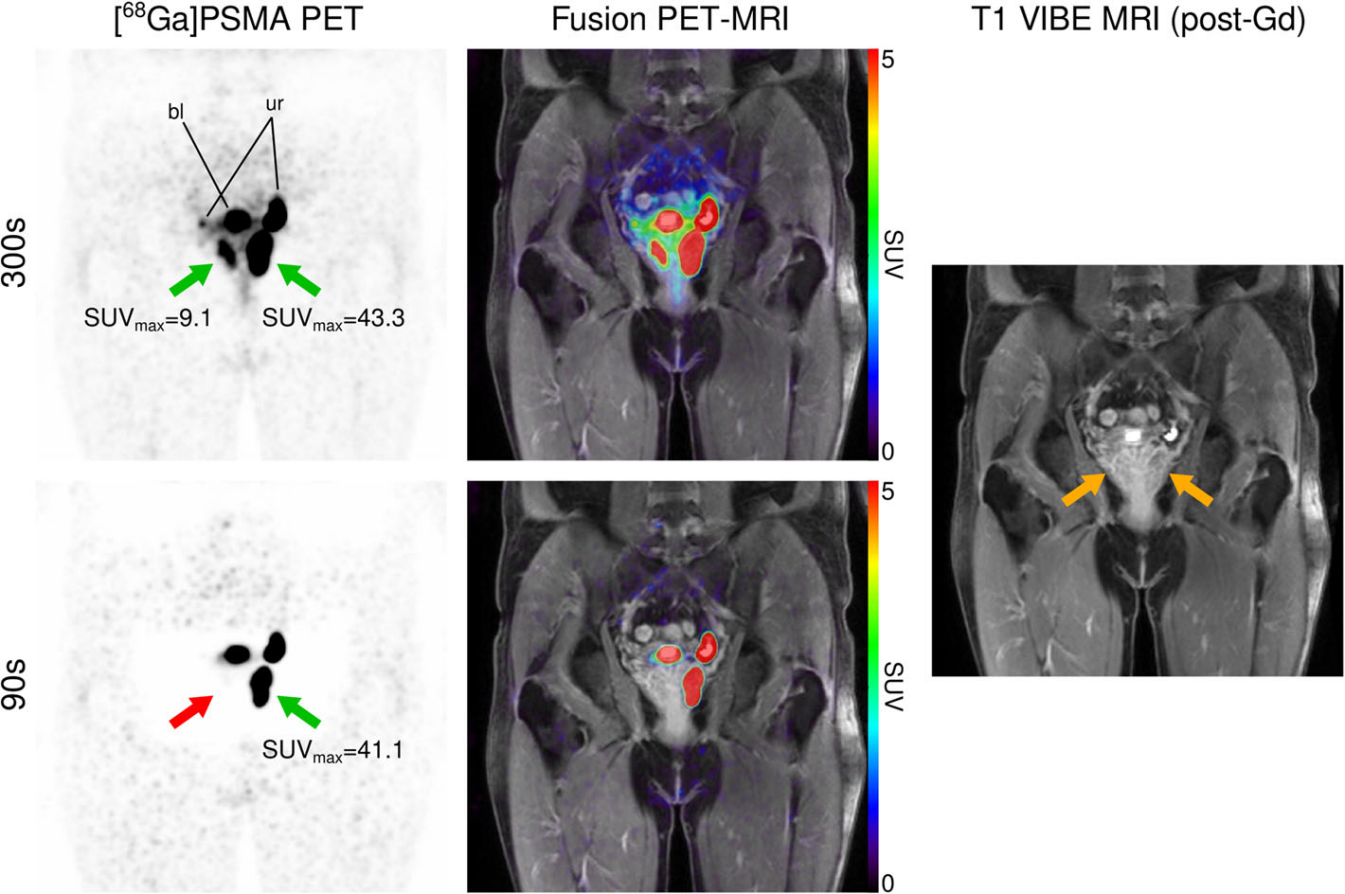
Coronal pelvic slice of a ^68^Ga-PSMA PET-MRI study dissecting the prostate gland, demonstrating loss of diagnostic accuracy at different PET acquisition durations per bed position (300 vs. 90 s). Green arrows refer to strong focal uptake in the prostate, highly suspicious for prostate cancer, while the *red arrow* refers to a missed lesion due to extensive halo artefact occurrence at 90 s. *Yellow arrows* denote lesion locations on the T1-weighted MR image. “bl”: urinary bladder; “ur”: ureter

Figure 8 demonstrates the impact of different acquisition durations in a clinical ^68^Ga-PSMA PET-MRI study of a 49-year-old patient (body mass: 79 kg; 159 MBq radiotracer; acquisition start 65 min post injection) who was referred to preoperative staging due to highly suspicious prostate exam.

At 300-s acquisition duration scatter-corrected PET showed intense tracer uptake of the entire left peripheral zone (SUV_max_: 43.3) and a focal uptake in the right peripheral zone (SUV_max_: 9.1) of the prostate gland, highly indicative for prostate cancer. Reduction of acquisition time to 90 s resulted in effacement of detectable tracer accumulation in the right peripheral prostate gland due to the presence of large halo artefact areas around the bladder.

## Discussion

The aim of this study was to evaluate the interrelation of PET acquisition time, attenuation correction, halo artefacts, and resulting lesion detectability in whole-body ^68^Ga-PSMA PET-MRI.

Results from this study show that halo artefacts around the kidneys and the urinary bladder, organs with high radiotracer activity, are indeed a relevant issue in ^68^Ga-PSMA PET-MRI acquired with the clinical Siemens mMR hybrid PET-MRI system, potentially hampering lesion detectability and quantification. Halo artefacts were found in approximately 40 to 90% of attenuation- and scatter-corrected images, depending on the acquisition time per bed position, going from 300 s down to 30 s. A similar proportion of patient scans with halo artefacts was previously reported by Afshar-Oromieh et al. They found artefacts around the kidneys in 11 and around the bladder in 15 out of 20 patients in scans with durations of 300 s per bed position [9]. However, since image acquisition was performed after completion of PET-CT and therefore much later than in our study, results are not comparable head-to-head.

The above data show that reduction of acquisition duration results in an increase in likelihood of halo artefacts in our setup, especially with durations lower than 180 s per bed position. In a recent publication by Lütje et al. the effect of acquisition time on the occurrence of artefacts has been studied for a single-bed position (pelvis) acquired in list mode approximately three hours after injection of ^68^Ga-PSMA tracer in PET-MRI [13]. Their results are largely in line with the results obtained within this study. However, the study presented here extends to whole-body acquisitions (instead of single-bed position scans) and a 1-h post injection imaging time point. Most importantly, we also studied potential causes for the observed acquisition time dependency of halo artefacts.

Since halo artefacts are not observed in images reconstructed without scatter correction and also not frequently encountered in PET from PET-CT [9], inadequate scatter correction, which in turn relies on the determined attenuation map, is a likely contributor to these artefacts. In order to further investigate this topic, we separated the effects of acquisition time reduction for emission data and for determination of the attenuation map in parts of the arms using the MLAA algorithm. Acquisition times were varied for one component at a time while leaving the other unchanged at 300 s. Results revealed that a reduction of acquisition time for the attenuation map resulted in more frequent and more severe halo artefacts, whereas a sole reduction of emission data statistics did not result in an increase of halo artefacts. Naturally, image noise increased for images with lower count statistics. This demonstrates at least one causative role of insufficient arm completion in the MLAA attenuation maps in the emergence of halo artefacts. In order to further investigate the role of MLAA arm completion, we investigated differences in the resulting attenuation map when varying the duration of data acquisition. Indeed, the volume of added arm attenuation not covered by the MR-derived attenuation map varied significantly with acquisition duration. Furthermore, the resulting density of the complemented arms in the attenuation maps varied considerably between acquired data with different durations. In conclusion, varying acquisition times per bed position not only directly influence the emission data but also heavily influence the attenuation map and further the scatter correction, specifically the scaling of the determined scatter distributions to scatter tails outside the assumed body contours.

^68^Ga also emits prompt gamma photons of 1077 keV in addition to positrons in a small amount of decays (≈1.2%), therefore potentially contaminating emission data when Compton-scattered prompt gamma photons are detected in coincidence with annihilation photons [19]. This contamination was shown to be at least in part responsible for introducing halo artefacts in ^68^Ga-PSMA PET-CT data, and dedicated correction methods resulted in PET images of reduced halo artefacts [17]. Our study demonstrates that the situation in PET-MRI seems to be different, as the positive impact of PGC was rather limited in terms of artefact occurrence. Possibly the influence of insufficient arm completion on halo artefacts is more important than prompt gamma contamination in PET-MRI, while prompt gamma contamination becomes a dominating cause for artefacts in PET-CT where CT-derived attenuation maps usually do not show truncation effects.

This is to our knowledge the first investigation of underlying mechanisms of ^68^Ga-PSMA-related artefacts in the Siemens Biograph mMR system for clinical whole-body scanning. As has been shown before [13], halo artefacts are clinically relevant as they may result in the non-detection of tumour lesions. Our results confirm this observation. We investigated the detectability of lesions and also their intensity. Lesions started to be missed in relation to the full 300-s scans when acquisition times per bed position fell below 180 s. Since halo artefacts do not appear in non-scatter-corrected images it has been proposed to compensate by additionally evaluating the non-scatter corrected images [13]. To test whether this approach is sufficient to alleviate the problems of halo artefacts in clinical routine imaging we analysed the lesion detectability of scatter-corrected and non-scatter-corrected images in various settings. For scan durations of 300 and 180 s per bed position, no relevant differences in lesion detectability were observed. Further reduction to 90 or 30 s frames led to a missing of lesions, even when analysing scatter-corrected and uncorrected images together. This demonstrated that analysing the non-scatter-corrected images cannot fully compensate the detrimental effects of the insufficient attenuation map at 90-s acquisition duration. It is a key result from this study that scan durations to be used for PET-MRI imaging cannot be taken from experience in PET-CT. In recent publications on ^68^Ga-PSMA PET-CT, acquisition times per bed position ranged from 5 min [9] to 1.5 min [20–22], the latter being proven too short for adequate imaging in PET-MRI in the settings of our study.

In this study we also analysed the impact of acquisition duration on standardized uptake values. Earlier studies using PET-CT have shown an increase of SUV_max_ with decreasing scan time due to increasing noise [23, 24]. We could also show this effect when reconstructing the images with reduced emission data and the full 300-s attenuation map. However, reconstructing the images with reduced statistics also used for generation of the attenuation map resulted in a drop of mean SUV_max_ at clinically used acquisition durations of 180 and 90 s. This effect is most probably caused by the insufficient arm completion and the resulting emergence of halo artefacts, overcompensating the effect of increasing image noise.

### Limitations

The results of this study strictly seem only valid for the setup of this study with regard to the imaging device (Siemens Biograph mMR), injected activity and delay between tracer injection and acquisition. However, since these conditions are characteristic for clinical imaging, the results should influence current PET-MRI imaging protocols, especially regarding acquisition duration per bed position.

If the injected activities were reduced or image acquisition would be started later after tracer injection, similar reductions in imaging accuracy as seen in this study would be expected. ^68^Ga-PSMA activities as low as ~50 MBq, which were shown to be sufficient for PET-CT imaging [25], might not suffice for current PET-MRI imaging. Lütje et al. have found an optimal imaging time of 4 min per bed position when imaging 3 h post-injection. This suggested longer acquisition duration as compared to ours is explainable by the later scanning time and the single-bed position scans, and hence lower sensitivity and count rates, and is thus in line with the results of this study with imaging one hour post injection. Furthermore, previous studies have shown a benefit on tumour-to-background ratios when postponing the acquisition start to 3 h after injection in PET-CT [26]. However, keeping in mind the effect of reduced soft tissue counts on the MLAA-based arm completion, this might be uncertain in PET-MRI imaging and imaging time will have to be longer to compensate for this effect.

Recently, novel PSMA ligands labelled with ^18^F instead of ^68^Ga have been developed (e.g., [^18^F]DCFPyL, [^18^F]PSMA-1007, and [^18^F]YC-88). It remains to be seen if these tracers will suffer from similar problems in clinical PET-MRI settings. However, one advantage besides others is the fact that ^18^F is a pure positron emitter without additional prompt gamma emission, thus avoiding halo artefacts introduced by this specific effect. Probably more important for image quality in PET-MRI is the biodistribution of these tracers, specifically the accumulation of radioactivity in the kidneys and the bladder. In this context, recent investigations demonstrated promising results for [^18^F]PSMA-1007 [27] and [^18^F]YC-88 [28], showing comparatively low uptake in the kidneys. These tracers might therefore be well suited for usage in current clinical PET-MRI systems.

## Conclusions

Halo artefacts around organs with high tracer uptake such as kidneys and urinary bladder are frequent in ^68^Ga-PSMA PET-MRI and related to acquisition duration per bed position when using clinically available scatter correction techniques. The use of emission data to fill in missing information about parts of the arms in the MR-derived attenuation maps seems to be an important mechanism for the occurrence of halo artefacts, so that minimum acquisition times cannot be transferred from PET-CT to PET-MRI. Acquisition times of 90 s per bed position or less lead to a lower detectability of lesions, caused by image noise and halo artefacts, that cannot be fully compensated by additional analysis of non-scatter-corrected images.

Acquisition times per bed position of at least 180 s are therefore recommended when using MLAA and injected tracer activities as in this study and a scan start one hour after injection.

## Additional files

Additional file 1: Halo scores for different acquisition durations and PET reconstructions. (ODS 10 kb)
Additional file 2: Lesion detectabilities for different acquisition durations and PET reconstructions. (ODS 13 kb)
Additional file 3: Lesion SUV_max_ detectabilities for different acquisition durations and PET reconstructions. (ODS 19 kb)
Additional file 4: Halo artefact occurrence vs. SUV_mean_ of high-contrast organs. (ODS 10 kb)
Additional file 5: MLAA-based arm completion for different acquisition durations. (ODS 11 kb)

## Abbreviations

AM: Attenuation map
CT: Computed tomography
MLAA: Maximum likelihood reconstruction of attenuation and activity
MRI: Magnetic resonance imaging
PET: Positron emission tomography
PGC: Prompt gamma correction
PSMA: Prostate-specific membrane antigen
SC: Scatter correction
SPECT: Single-photon emission computed tomography
SUV: Standardised uptake value
